# Protein crystal screening and characterization for serial femtosecond nanocrystallography

**DOI:** 10.1038/srep25345

**Published:** 2016-05-03

**Authors:** Connie Darmanin, Jamie Strachan, Christopher G. Adda, Thomas Ve, Bostjan Kobe, Brian Abbey

**Affiliations:** 1 ARC Centre of Advanced Molecular Imaging, Department of Chemistry and Physics, La Trobe Institute for Molecular Science, La Trobe University, Melbourne, Victoria 3086, Australia; 2 Department of Biochemistry and Genetics, La Trobe Institute for Molecular Science, La Trobe University, Melbourne, Victoria 3086, Australia; 3 School of Chemistry and Molecular Biosciences and Institute for Molecular Bioscience (Division of Chemistry and Structural Biology) and Australian Infectious Diseases Research Centre, University of Queensland, Brisbane, 4072, Australia; 4 Institute for Glycomics, Griffith University, Gold Coast Campus, Southport, QLD, 4222, Australia; 5 Melbourne Centre for Nanofabrication, Melbourne, 3168, Australia

## Abstract

The recent development of X-ray free electron lasers (XFELs) has spurred the development of serial femtosecond nanocrystallography (SFX) which, for the first time, is enabling structure retrieval from sub-micron protein crystals. Although there are already a growing number of structures published using SFX, the technology is still very new and presents a number of unique challenges as well as opportunities for structural biologists. One of the biggest barriers to the success of SFX experiments is the preparation and selection of suitable protein crystal samples. Here we outline a protocol for preparing and screening for suitable XFEL targets.

X-ray free electron lasers (XFELs) are the latest cutting-edge tool for structural biologists. The highly intense, femtosecond pulse structure make them ideally suited to the study of nanocrystals and for investigating the dynamics of proteins. Currently, there are two hard X-ray free electron lasers in operation that can be used for serial femtosecond crystallography (SFX); these are the Linac Coherent Light Source (LCLS) in the USA and the SPring-8 Angstrom Compact Free Electron Laser (SACLA) source in Japan. The unique capability of these sources and their scarcity mean that there is a constant high-demand for access to these facilities. Recent XFEL experiments have resulted in a number of high-impact biological structures, which include but not limited to the photosystem I and the serotonin receptor^1^,^2^,^3^,^4^,^5^,^6^,^7^,^8^. This technology, however, is still in its infancy and little has been published regarding the preparation and selection of suitable XFEL crystalline samples, which differ, in many cases significantly, from the types of crystals that are routinely measured at synchrotron sources.

Significant advancements in micro-focus crystallography beamlines at third-generation synchrotrons have seen the size of crystals that can be measured at such sources become ever smaller. The minimisation of background signal and the introduction of serial synchrotron crystallography (SSX) has extended the limit from which single crystal data can be collected at the synchrotron to the micron scale^9^,^10^. For protein crystals well-below the 1 micron scale, (i.e. those where growth is difficult to optimise beyond the nanometre scale), SFX is the only viable option for obtaining high-resolution single crystal diffraction data due to the rapid degradation of the measured diffracted intensities by onset radiation damage^11^.

Even with the extremely intense beams available at XFELs, measuring samples with the correct characteristics in terms of size, quality and crystalline order is essential for a successful experiment. Nanocrystals that are poorly ordered will likely not yield high-resolution at the XFEL, whereas those that are highly ordered will still be too small for single crystal synchrotron measurement but may be perfect candidates for XFEL analysis. The key questions that need to be answered in screening for suitable XFEL targets therefore are:

1. Is the crystal too small for single crystal measurements at the synchrotron?
2. Is the crystal protein or salt?
3. Is the crystal quality sufficient for high-resolution structural information to be collected at the XFEL?

As we normally expect to work with nanocrystals that are below the size range of many standard protein crystal characterization techniques – answering these questions is a non-trivial exercise and requires the development of new protocols specific to nanocrystals. Currently, methods for looking at diffraction quality of nanocrystals and visualising nanocrystals are being developed^12^,^13^. However, here we describe a method that we have developed using a combination of standard laboratory and synchrotron-based techniques for selecting appropriate nanocrystallography targets that allows identification of crystal size and crystal quality checks to see if they are suited for XFEL experiments or require further crystallization optimization.

In order to demonstrate the effectiveness of the protocols we have developed for SFX screening, we present an example case study carried out on a simple test systems: lysozyme. In a ‘blind test’, we then apply this protocol to a new protein target, MAL TIR domain-induced MyD88 TIR domain (hereafter MyD88^TIR^) crystals. We demonstrate that the protocol we outline is able to generate sufficient data to determine that this sample is suitable for XFEL experiments. Our approach combined data from a variety of biophysical characterisation techniques including light microscopy, transmission electron microscopy (TEM), dynamic light scattering (DLS) and synchrotron radiation.

## Results

### Crystal imaging and size determination

#### Microscopy

**Figure 1:**
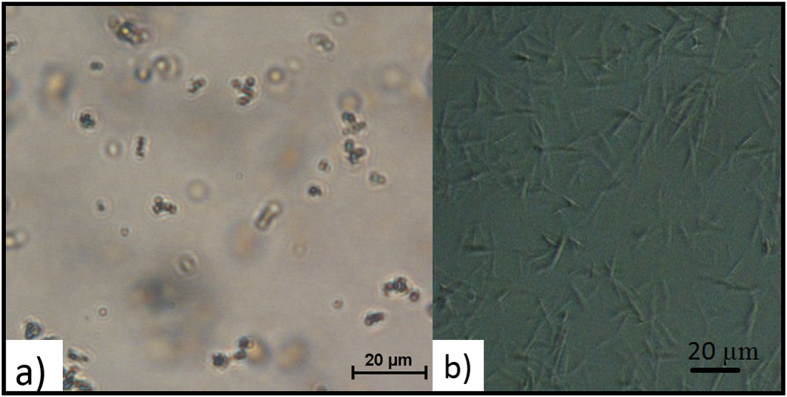
Images of crystals using an optical microscope. (**a**) Lysozyme and (**b**) MyD88^TIR^ crystals. The estimated size of the lysozyme crystals were ~2 μm. MyD88^TIR^ crystals were rod-shaped and the estimated sizes ranged from ~10 μm to 20 μm in the longest dimension. These images were taken using a Nikon- ‘Eclipse Ti-s’ optical microscope.

The optical microscope is a useful tool that is routinely used to view protein crystals and obtain an estimate of their size. However, for crystals that are below the size range of conventional protein crystals (<5 μm), the use of an optical microscope can be challenging. Figure 1a shows an image of lysozyme crystal with sizes around 2 μm. A sharp image was hard to obtain using the highest magnification (x100) of the Nikon optical microscope due to the limitation of the microscope itself. Crystals of the MyD88^TIR^ samples could not be clearly seen under the light microscope however the morphology of the rod shapes could be identified (Fig. 1b).

**Figure 2:**
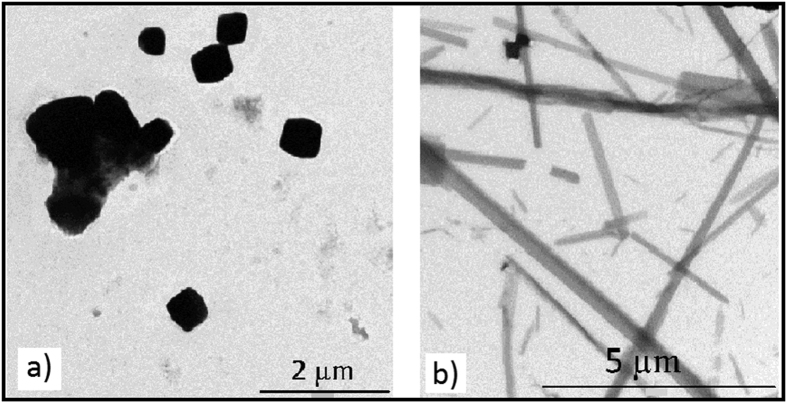
Negative stained TEM images of protein crystals. (**a**) Lysozyme tetrahedral crystals and (**b**) MyD88^TIR^ rod-shaped crystals. The crystals were taken from the same sample as in Fig. 1. The crystals were stained with 1% uranyl acetate and visualised in a JEOL JEM-2010 TEM.

Transmission electron microscopy (TEM) was used to obtain better images of the crystals. TEM revealed the crystallization slurry had a mixed crystal population for both samples tested. The lysozyme crystal morphology was tetrahedral and ranged from 2 μm to 50 nm in size (Fig. 2a). The MyD88^TIR^ sample showed rod-shaped crystals ranging from 12–0.5 μm in the longest dimension x ~0.1 μm (Fig. 2b).

#### Dynamic light Scattering (DLS)

**Figure 3:**
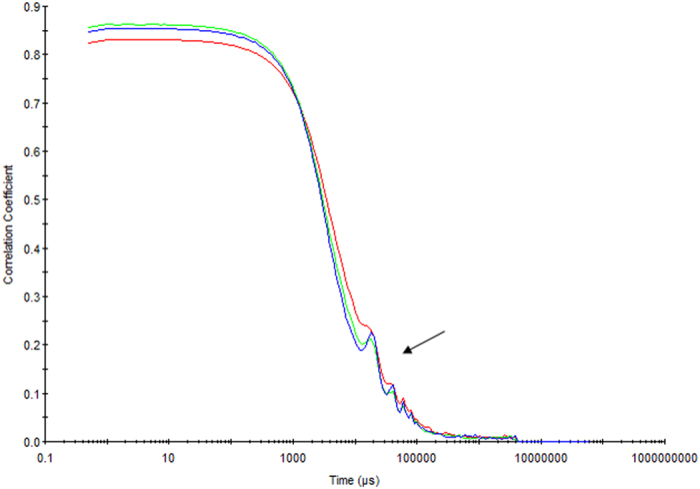
Representative correlation graph for the dynamic light scattering results of a slurry of MyD88^TIR^ crystals filtered through a 10 μm frit. Each coloured line represents a different sample run and the peaks highlighted by the arrow indicate the sample is heterogeneous.

The size of the crystals in solution was also tested using DLS. Table 1 show a number of individual runs for the DLS results of a crystallization slurry for lysozyme and MyD88^TIR^ crystals. The estimated size of the particles in solution for lysozyme corresponded to 2.3 ± 0.6 μm, with a poly-dispersity index (PDI) of 0.4 ± 0.25 (from an average of 4 runs). The estimated size of MyD88^TIR^ crystals from an average of three runs corresponded to 3.9 ± 0.3 μm, with an average PDI of 0.408 ± 0.1 (Refer to Supplementary Information, Figure S1–3 for individual measurements). The DLS results confirmed the heterogeneity of the samples observed in the TEM images (Fig. 2), as indicated by the multiple peaks seen in the correlation data graph (Fig. 3). The reliability of this data for crystalline samples must be taken with caution as DLS is a technique not well suited for crystalline samples. Therefore this data only provides an estimate of crystal size of the dominant species in the sample and is not a true value of crystal size.

#### Synchrotron crystal quality characterization

**Figure 4:**
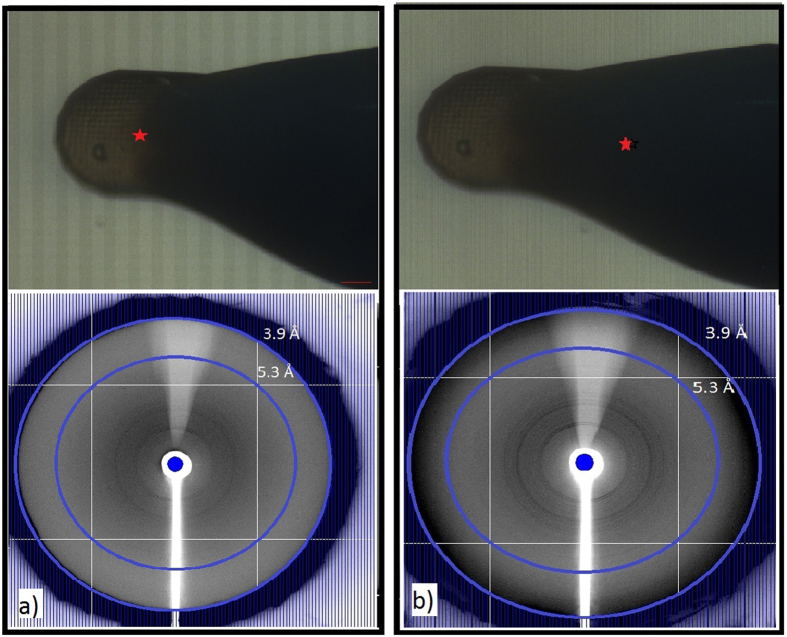
Images of the sample loop and the synchrotron X-ray diffraction of MyD88^TIR^ crystals, showing the effect of increasing the number of crystals in the X-ray beam. The position of the X-ray beam is indicated by the red star. (**a**) The X-ray beam is positioned on a low density of crystals in the loop and shows weak diffraction, ~8.5 Å resolution. (**b**) The sample is moved across to the middle of the loop so that the X-ray beam is focused on a higher density of crystals and shows diffraction to ~5.8 Å resolution. The sample-to-distance was 400 mm with an exposure time 5 seconds. The blue rings indicate the resolution rings.

To verify if the crystal quality was sufficient for high-resolution structural information to be collected at the XFEL, we obtained preliminary X-ray diffraction data using a synchrotron source. A small amount of poly-crystalline sample was placed in the X-ray beam and a diffraction image was taken. We continued to increase the amount of crystals in the X-ray beam to see how it affected the diffraction pattern. Figure 4 shows a photo of the MiTeGen loop loaded with a low number of crystals (Fig. 4a) followed by a larger number of crystals (Fig. 4b). Due to a large number of crystals present on the loop, a poly-crystalline powder diffraction image was observed (Fig. 4). As we increased the volume of crystals in the X-ray beam an increase in diffraction resolution of the sample was observed. This is demonstrated in Fig. 4 for the MyD88^TIR^ crystals. Similar results were also seen for the lysozyme crystals.

**Figure 5:**
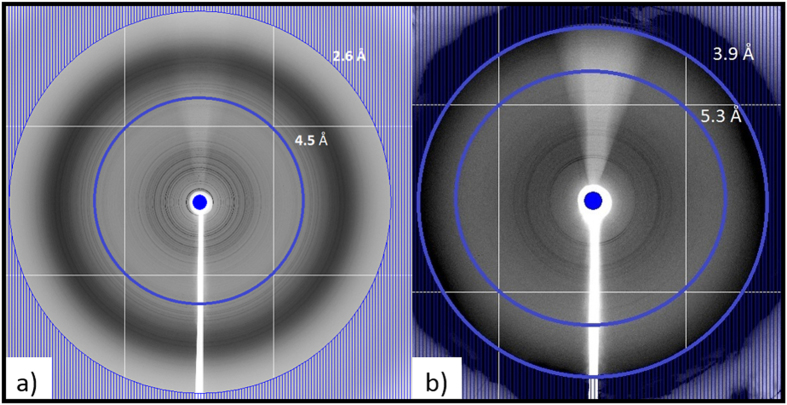
X-ray diffraction images of a slurry of crystals placed in the synchrotron X-ray beam. (**a**) Lysozyme crystal diffraction to 2.6 Å and (**b**) MyD88^TIR^ crystal diffraction to 5.6 Å resolution. Data was collected at the Australian Synchrotron (MX2 beamline) and the blue rings indicate the resolution rings.

Figure 5 shows the polycrystalline diffraction pattern resulting from a large number of crystals placed in the X-ray beam for lysozyme (Fig. 5a) and MyD88^TIR^ (Fig. 5b) crystals. Lysozyme and MyD88^TIR^ crystals diffract to 2.6 Å and 5.6 Å, respectively. The raw data indicates that the MyD88^TIR^ crystals may diffract beyond 5.6 Å as indicated by very faint powder rings which cannot be seen in these images.

## Discussion

Crystal characteristics such as size, quality and crystalline ordering will determine the success of XFEL experiments. Nanocrystals that are poorly ordered are unlikely to yield high-resolution data at the XFEL, whereas those that are highly ordered but too small for single crystal synchrotron measurements, may be perfect candidates for XFEL analysis. Imaging crystals to get an indication of size and quality is critical for successful serial femtosecond nanocrystallography (SFX) experiments. Knowing the crystal size helps determine parameters for the jet delivery system and data collection setup. The crystal size will affect which is the nozzle size and the initial beam diameter for data collection. Whether the crystals are clumping; What shape the crystals are; and What size the crystals are; represent some relevant questions that need to be answered prior to XFEL experiments? Nano-crystals are difficult to image using an optical microscope, which is evident in our results. Optical microscopy is able to provide an estimate of crystal sizes above approximately 2 μm, but it is difficult to see if the sample is homogenous. TEM provides an accurate measure of crystal size. It also provides an indication of crystal morphology and size distribution within the sample. We have shown DLS was able to provide a good estimate of crystal size and an indication of sample heterogeneity. Therefore, we found that a combination of three complementary techniques, optical microscopy, TEM and DLS, is able to provide an overview of the crystal size and morphology in our sample. The morphology and the shape of the MyD88^TIR^ crystals allows us to determine the starting experimental parameters for the XFEL beam-time, which includes the beam diameter and ideal nozzle size to use. The identification of the rod-shaped morphology of the crystals allows experimental planning to help design an experimental setup for data collection. Due to their morphology, we would assume the crystals will have preferential orientation (in the direction of their longest dimension) when exiting the GVDN jet-stream. Therefore we have to take into the effect of the flow alignment of the rod-shaped crystals and optimise the jet delivery rate for this sample accordingly.

Synchrotron characterization showed that the crystal size was too small to carry out conventional single-crystal diffraction at the synchrotron source, and therefore is an ideal candidate for XFEL. Using the synchrotron data, we are able to help determine the crystal composition (i.e. protein or salt, as indicated by the spacing of the diffraction rings seen in diffraction images (large spacing between the powder rings is indicative of a small molecule, while small spacing is indicative of a large molecule). The synchrotron data also allows us to access the crystal quality.

Crystal quality is important for determining if the sample is suitable for obtaining high-resolution structural information at the XFEL. The first assumption that most people, who are new to the field of XFEL science make, is that poor diffraction from crystals using a synchrotron source will lead to better diffraction using a highly focused XFEL beam. This may not be the case, as it is highly dependent on the quality of crystals in the sample. A crystal that has poor crystalline packing and does not diffract well at a synchrotron, will not diffract any better at the XFEL, due to the intrinsic disorder of the crystal. Determining the differential effects of poor quality nanocrystals and not enough photons on the sample can be difficult. We have demonstrated a method, using a synchrotron radiation source that can determine if the X-ray diffraction of the crystal is limited due to the intrinsic disorder of the crystal or the lack of photons generated by the source. Increasing the numbers of crystals in the X-ray beam resulted in an increase in resolution of the diffraction pattern in the image. This is a direct result of the crystals magnifying the Bragg reflections and is indicative that the crystal quality of the sample is well ordered; therefore, high resolution data may be restricted by the photon flux of the synchrotron rather than the crystal quality. This was clearly demonstrated for the two crystal systems tested, especially the MyD88^TIR^ crystals, which were the smallest crystals tested. If the crystals contained intrinsic disorder, we would not expect an increase in resolution of the sample in the diffraction image as we increased the number of crystals in the X-ray beam. This is because the disorder of the crystals would remain the same no matter how many crystals were present.

Crystal size distribution in the sample will also affect the data quality. Better diffraction data can be collected if the average crystal size is comparable to the width of the focal point^14^. A large variation in the crystal size in the sample will therefore affect the quality of data. Currently, the successful hit rates for SFX can be as low as 4.8% of the total hits^15^, due to a combination of collecting data from poorly diffracting crystals and air scatter (images where no crystal are present). Our results show that both samples investigated have heterogeneity in the crystal population size. Further characterization and separation of the crystal populations within the sample would be advantageous for data collection, to improve the quality of the sample and increase the successful hit rate for SFX. This will be a focus of a separate publication.

Overall, we have developed a method using standard laboratory and synchrotron-based techniques for selecting appropriate nano-crystal targets for XFEL experiments. Characterization of the MyD88^TIR^ crystals have provided substantial evidence to support the case that the crystal are well ordered and suitable for SFX at an XFEL. The experimental characterization results show that a combination of optical microscopy, transmission electron microscopy, dynamic light scatter and synchrotron data we were able to determine the crystal size, morphology, sample heterogeneity and diffraction quality for crystals that are difficult to see optically. These results, in combination with the fact that we can grow large volumes of these crystals, makes the MyD88^TIR^ crystals ideal for serial femtosecond nanocrystallography experiments at an X-ray free electron laser.

## Methods

Lysozyme (L6876-10G) was purchased from Sigma-Aldrich. All chemicals used were purchased from Sigma-Aldrich unless otherwise stated. 24-well crystallization plates were purchased from Hampton Research and ZEN0112 UV disposable cuvettes used for DLS experiments were purchased from ATA Scientific. Crystallography meshes were purchased from MiTeGen.

**Figure 6:**
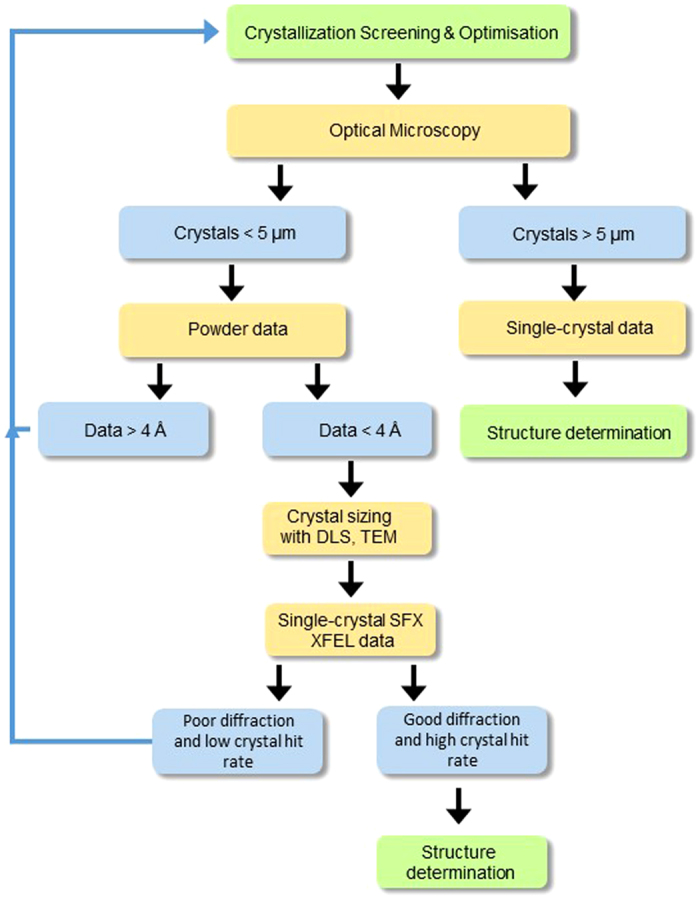
A flow diagram showing the overall process for sample characterization, starting from a crystallized sample to obtaining experimental justification for an XFEL serial femtosecond nano-crystallography experiment (SFX). Yellow boxes corresponds to the data analysis steps, green boxes correspond to the characterization steps and blue boxes correspond to the ‘decision making’ steps. DLS; dynamic light scattering, TEM; transmission electron microscopy.

The samples were first crystallized and imaged using several techniques to determine the crystal size. The diffraction quality of the crystals was tested on the Australian Synchrotron. A flow diagram of the method developed is shown in Fig. 6 and the following section outlines the method in more detail.

### Crystallization

#### Lysozyme crystallization

Lysozyme crystals were grown based on a protocol adapted from Falkner *et al*.^16^. The crystals were grown using the batch crystallization method in 1.5 ml centrifuge tubes. 200 μl of crystallization solution (18% sodium chloride, 6% PEG 6000, pH to 4 using sodium hydroxide) was added to 20 μl of 160 mg/ml lysozyme protein made up in 0.5 M acetic acid (pH 4, using sodium hydroxide), and vortexed immediately. The crystallization reaction was sealed closed and placed in a 22 °C incubator. Crystals grew spontaneously in solution and equilibrated overnight.

#### Production of MAL induced MyD88 TIR domain crystals

The TIR domains of human MAL (residues 79–221 with a N-terminal His_6_-tag) and MyD88 (residues 155–296 with a C-terminal His_6_-tag) were produced in *E. coli* BL21 (DE3) cells using the auto-induction method^17^, and purified using a combination of immobilized metal ion affinity chromatography and gel-filtration. For the gel-filtration step we used a Superdex 75 HiLoad 26/60 size-exclusion chromatography (SEC) column (GE Healthcare) pre-equilibrated with buffer containing 10 mM HEPES (pH 7.5) and 150 mM NaCl. MAL induced MyD88 TIR domain crystals were produced by incubating MAL TIR domain (0.5–3 μM) with MyD88 TIR domain (60 μM) in 10 mM HEPES pH 7.5, 150 mM NaCl at 25–37 °C for 1–2 hours.

### Crystal Imaging and Size Determination

#### Optical microscopy

A Nikon, ‘Eclipse Ti-s’, optical microscope was used to image and determine the size of the crystals. 2 to 5 μl of crystallization slurry was placed on a cover slip and inverted onto a microscope slide to image the crystals.

#### Transmission electron microscopy (TEM)

The samples were prepared on a carbon-coated 400 mesh copper grids (ProSciTech, GCU400H). A 4 μl aliquot of a 1 in 1000 dilution of crystals was placed on a grid and allowed to stand for 2 minutes. The excess solution was drawn off using filter paper and 4 μl of 1% uranyl acetate was added directly on top of the grid and drawn off immediately using filter paper. The sample was left to air dry for 10 minutes before imaging in a JEOL JEM-2010 transmission electron microscope operated at 100 kV. Images were taken using a Valeta 4 MP CCD camera.

#### Dynamic light scattering

The average particle size of crystals in the crystallization slurry were determined using a Malvern Zetasizer Nano-ZS dynamic light-scattering (DLS) analyser (Malvern Instruments Ltd., Malvern, Worcestershire, UK). A 40 μl aliquot of diluted crystals was analysed for particle size in multiple runs. The particle size reported presented hydrodynamic diameters derived from the time-correlation function of the particle number density^18^. The MyD88^TIR^ crystals were pre-filtered through a 10 μm frit prior to DLS analysis to discard any crystals aggregates from the sample.

### Synchrotron crystal quality characterization

#### Synchrotron crystal sample preparation

A 50 μl aliquot of crystal slurry was centrifuged at 2000 × *g* for 5 min at room temperature to pellet the crystals. The liquid surrounding the pellet was aspirated off and replaced with a cryo-solution and re-centrifuged at 2,000 × *g* for 5 min to pellet the crystals. Most of the supernatant was removed from the crystals. A large volume of the crystals was pipetted onto a MiTeGen mesh and plunged into liquid nitrogen and placed on a standard crystallography mount at the MX2 beamline at the Australian Synchrotron.

All crystals were placed in cryogenic solution; MyD88^TIR^ crystals contained 16% glycerol, and lysozyme crystal contained 25% PEG400.

#### Data Collection

The diffraction images were obtained at the MX2 micro-focus beamline at the Australian Synchrotron. A beam with a wavelength of 0.957 Ǻ (13.0 keV) and dimensions 25 μm × 15 μm was used with 5 to 10 second exposure time, no attenuation and a typical X-ray flux of 5 × 10^12^ photons/s. Data was collected at detector-to-sample distance of 500 mm for MyD88^TIR^ crystals, and 400 mm for lysozyme crystals. 2–D diffraction images were recorded on an ADSC Quantum 315r CCD. All images were viewed using iMosflm^19^.

## Additional Information

**How to cite this article**: Darmanin, C. *et al*. Protein crystal screening and characterization for serial femtosecond nanocrystallography. *Sci. Rep*. **6**, 25345; doi: 10.1038/srep25345 (2016).

## Supplementary Material

Supplementary Information

## Figures and Tables

**Table 1:** Z-Average particle size and poly-diverse index (PDI) for the size intensity distribution plots shown in Figure S1 and Figure S2 for Lysozyme and MYD88^TIR^ crystals respectively.

Sample	Z-Average Size (nm)	PDI
Lysozyme Run 1	1708	0.678
Run 2	2568	1
Run 3	2518	0.082
Run 4	2762	0.01
MYD88^TIR^ Run 1	4273	0.433
Run 2	3606	0.300
Run 3	3875	0.491
