# Supplementary material for: Protein crystal screening and characterization for serial femtosecond nanocrystallography
Source: Sci Rep. 2016 May 3;6:25345. doi: 10.1038/srep25345 (PMC4853777; doi:10.1038/srep25345)
Supplement: Supplementary Information [file srep25345-s1.pdf]

Protein crystal screening and characterization for serial femtosecond nanocrystallography

Connie Darmanin, Jamie Strachan, Christopher G. Adda, Thomas Ve, Bostjan Kobe, Brian Abbey

Supplementary information

Dynamic Light Scattering results

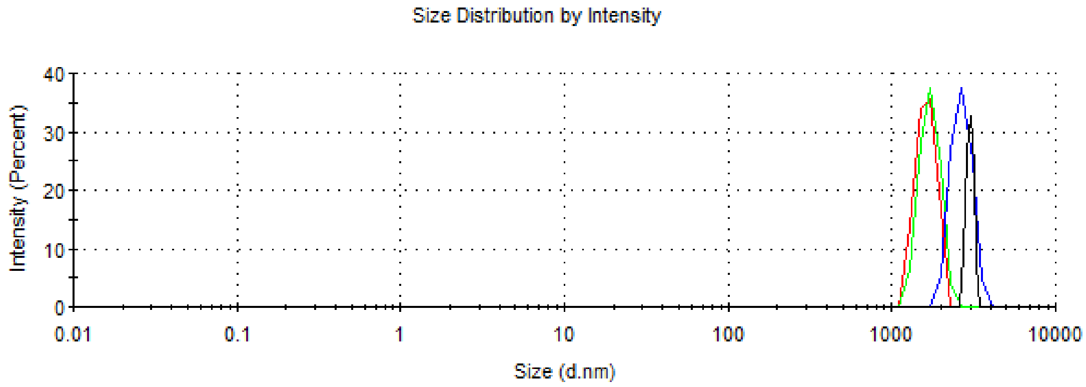

Figure S1: DLS intensity distribution plots for Lysozyme crystals. The Lysozyme crystals were tested four times and provided and average particle size of the Lysozyme crystals predominately persistent in the sample. Each colour represents a different run (red is run 1, green is run 2, blue is run 3 and black is run 4) for the same sample.

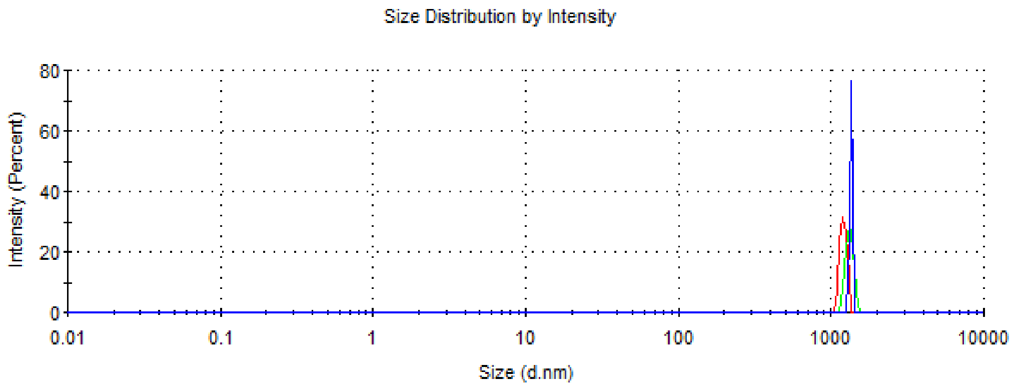

Figure S2: DLS intensity distribution plots for MYD88^{TIR} crystals. The MYD88^{TIR} crystals were tested three times and provided and average particle size of the MYD88^{TIR} crystals predominately persistent in the sample. Each colour represents a different run (red is run 1, green is run 2 and blue is run 3) for the same sample.

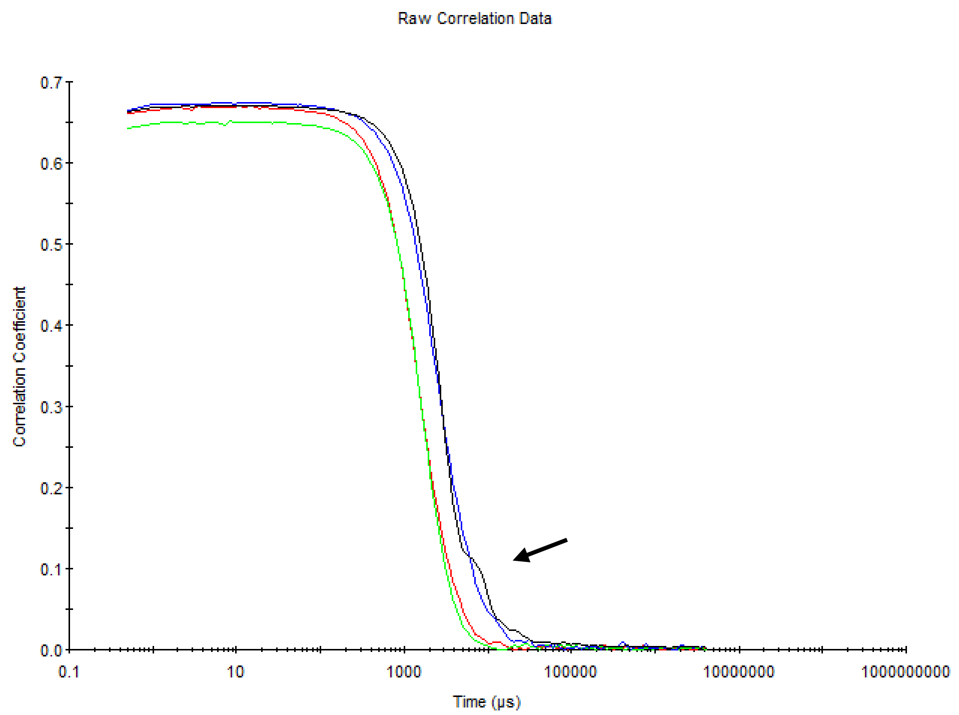

Figure S3: Correlation graph for the dynamic light scattering results of a slurry of Lysozyme crystals. Each coloured line represents a different sample run (red is run 1, green is run 2, blue is run 3 and black is run 4) and the peaks highlighted by the arrow indicate the sample is heterogeneous.
